# Histological and Ultrastructural Studies on the Conjunctiva of the Barred Owl (*Strix varia*)

**DOI:** 10.1371/journal.pone.0142783

**Published:** 2015-11-12

**Authors:** Brian Jochems, Thomas E. Phillips

**Affiliations:** 1 Division of Biological Sciences, University of Missouri, Columbia, MO, United States of America; 2 College of Veterinary Medicine, University of Missouri, Columbia, MO, United States of America; Justus-Liebig-University Giessen, GERMANY

## Abstract

This report is the first characterization of the histology and ultrastructure of the barred owl conjunctiva. The inferior eyelid was dominated by a large disk-shaped plate covered by a non-keratinized stratified squamous or cuboidal epithelium of variable thickness. The apical surface of the plate epithelium varied from flat to long microvilli or even short cytoplasmic extensions similar to those seen in the third eyelid. All specimens had a few goblet cells filled with mucous secretory granules in the plate region. The underlying connective tissue was a dense fibroelastic stroma. Eosinophils were surprisingly common in the epithelial layer and underlying connective tissue in the plate and more distal orbital mucosal region. The orbital mucosa contained goblet cells with heterogeneous glycosylation patterns. The leading edge and marginal plait of the third eyelid are designed to collect fluid and particulate matter as they sweep across the surface of the eye. The palpebral conjunctival surface of the third eyelid was covered by an approximately five-cell-deep stratified squamous epithelium without goblet cells. The bulbar surface of the third eyelid was a bilayer of epithelial cells whose superficial cells have elaborate cytoplasmic tapering extensions reaching out 25 μm. Narrow cytofilia radiated outwards up to an additional 15–20 μm from the cytoplasmic extensions. Lectin labeling demonstrated heterogeneous glycosylation of the apical membrane specializations but only small amounts of glycoprotein-filled secretory granules in the third eyelid.

## Introduction

Birds, especially owls, have eyes that are large compared to their body mass to allow them to meet the demands of rapid flight, prey detection, and night vision. The eyelids, including the third eyelid (membrana nictitans), contribute to the formation and spreading of the precorneal tear film and play an essential protective role in clearing debris from the surface and preventing desiccation of the tear film during flight [1]. Although the avian eyelid is known to have a distinctive morphology, histological characterization of the conjunctiva in birds of prey has not typically been done due to understandable restrictions on collection of tissues from these protected animals. A unique opportunity to survey the conjunctival histology of free-living barred owls (*Strix varia*) was made possible when we were given access to birds which had been recently euthanized due to unrecoverable injuries

The gross and microscopic anatomy of eyelids in raptor species such as owls and hawks has been well-described [2]. The inferior eyelid, being larger and more mobile than the superior eyelid, is able to cover the majority of the eye in the closed position. It has a large oval plate with a slightly concave surface that allows it to conform to the curvature of the cornea. The dense fibrous connective tissue underlying this region is similar to what would be found in the mammalian tarsal plate. Unlike the tarsal plate, however, this structure is only found in the inferior eyelid. Furthermore, no meibomian or other glands are found in in this region of the avian conjunctiva. To emphasize the differences with the mammalian tarsal plate region, we will adopt the terminology employed by Murphy and simply refer to this region as the inferior fibrous plate [2]. The histology of the fibrous plate’s superficial epithelium has been largely unstudied.

The third eyelid moves across the cornea in a dorsonasal to ventrotemporal direction in owls [1, 2]. In some avian species, the third eyelid has a unique epithelium with elongated cellular protrusions and extensive filaments projecting from its apical surface [3–6]. This so-called “feather-duster” or “feathered” epithelium distinguishes the avian third eyelid from all other conjunctivae and is arguably a unique epithelial morphological specialization found in no other location. Earlier microscopic descriptions of the avian third eyelid have primarily been studied using the pigeon, ducks or rockhopper penguin which are phylogenetically distinct from the land bird clade that includes the passerines and birds of prey such as owls, hawks, falcons, and vultures [4–8].

The elaborate epithelium of the third eyelid and unusual inferior plate are two of the most distinctive features of the barred owl eyelids and are the special focus of this paper.

## Materials and Methods

### Animals

Ocular tissues were collected from one juvenile and five mature barred owls immediately after euthanasia was performed on birds with terminal injuries (Table 1). The birds were animals that had been found injured in the wild and brought by members of the general public for care under the auspices of the Raptor Rehabilitation Project of the College of Veterinary Medicine of the University of Missouri. Despite the Raptor Rehabilitation Project having a success rate for rehabilitation and release that exceeds the national release rate for raptor rehabilitation centers, some birds arrive at the facility too injured to fully recover. All decisions regarding the need for euthanasia were made by individuals concerned solely with the humane treatment and care of the birds and who had no conflict of interest since they were not associated with the subsequent histological studies. The clinical history of the birds reported in Table 1 gives insight into reasons euthanasia was necessary. Tissues were collected under U.S. Fish & Wildlife Service Migratory Bird permit SCCK021187A (T.E.P). Since the tissues were collected from animals euthanized for reasons other than research, no additional protocol number was required for the work reported here under the policies of the University of Missouri Animal Care and Use Committee.

**Table 1:** Clinical history of birds used in this study.

Animal number	Weight (kg)	Clinical notes from medical records
1	0.82	Distal right tibiotarsal fracture possibly involving the joint, possible left proximal humerus fracture or shoulder luxation.
2	0.50	A proximal ulnar fracture and radial head luxation of the left wing.
3	0.68	Bilateral detached retinas and uveitis. Absence of trauma suggestive of systemic illness.
4	0.65	Obtunded, severely dehydrated despite treatment with a shock dose of hypertonic saline and 10% dehydration fluids, and diarrhea. Bilateral mild uveitis and insipient cataracts.
5	0.60	Open left humoral fracture over 1 week old that could not be corrected.
6	0.52	Juvenile < 4 months old, open fracture of right humerus

### Tissue processing for light microscopy

The entire eye and surrounding tissues were dissected bluntly and immersed in 2% paraformaldehyde in HEPES Wash Buffer (HWB: 70 mM NaCl, 30 mM HEPES, 2 mM CaCl_2_, pH 7.4) within 30 min of euthanasia. After a minimum of 24 hrs, the tissues were rinsed and further dissected. For routine histology, tissues were embedded in paraffin, sectioned and stained using hematoxylin and eosin. For lectin labeling studies, tissues were rinsed in 50 mM glycine in HWB to block free aldehyde groups, dehydrated with a series of increasing concentrations of ethanol, infiltrated by butyl-methylmethacrylate (BMMA) and then the resin was polymerized at 4˚C using ultraviolet light [9]. Semi-thin 0.5 μm BMMA sections mounted on glass slides were immersed in acetone for 10 min to remove the resin, rinsed thoroughly and blocked for 1 hr in 1.0% bovine serum albumin (BSA) in HWB. Sections were incubated overnight with 10 μg/ml of a biotinylated lectin plus 10 μg/ml of a fluorescein-labeled lectin followed by rinsing and incubation in 1 μg/ml streptavidin-Alexa568 in HWB + 0.1% BSA for 4 hrs. All lectins were purchased from Vector Laboratories (Burlingame, CA) and their full names, abbreviations and binding specificity are shown in Table 2. Nuclei were counterstained by adding 300 nM DAPI (4’,6-diamidino-2-phenylindole) to the streptavidin-Alexa568. In some cases, a cocktail of fluorescein-labeled lectins (WGA, SBA, DBA, GSL-I, and SNA) all at 10 μg/ml, was employed. Streptavidin-Alexa568 and DAPI were obtained from Life Technologies (Grand Island, NY).

**Table 2:** Lectins used in this study and their nominal binding specificity.

Lectin	Preferred Sugar Specicity
DBA *Dolichos biflorus* agglutinin	N-acetylgalactosamine (α > β) [10]
ECL *Erythrina cristagalli* lectin	Galactose β(1–4) N-acetylglucosamine [11]
GSL-I *Griffonia simplicifolia* lectin I	αGalactose, αN-acetylgalactosamine [12]
GSL-I-B4 *Griffonia simplicifolia* Isolectin B4	αGalactose [12]
LCA *Lens culinaris* agglutinin	αMannose, αGlucose [13]
LEL *Lycopersicon esculentum* lectin	N-acetylglucosamine oligomers and poly-N-acetyllactosamine (Gal β1,4 GlcNAcβ1,3)_n_ repeating units.[14, 15]
MAL-I *Maakia amurensis* lectin I	Sialic acid (α2–3) galactose [16]
SBA Soybean agglutinin	N-acetylgalactosamine (α > β) [13]
SNA *Sambucus nigra* agglutinin	Sialic acid (α2–6) galactose [17]
VVA *Vicia villosa* agglutinin	N-acetylgalactosamine [18]
WGA Wheat germ agglutinin	N-acetylglucosamine, sialic acid [13]

### Light microscopy image acquisition

The macro-view of the front of the eyelid was taken using the Leica Application Suite (LAS; (Leica Microsystems; Buffalo Grove, IL) software multifocus software option on a Leica M205FA stereoscope to acquire an image with an extended depth of focus. To improve the contrast in the melanocyte-rich region, images were collected using the high dynamic range option of the LAS software.

For the panoramic views of the conjunctiva, a series of overlapping brightfield images of paraffin sections was acquired using a 10x objective on a Leica DM5500B microscope equipped with a Leica DFC290 camera. The LAS software was used to create the composite image and the software’s shading correction option ensured a uniform background illumination during collection of the brightfield images. The resulting composites were pasted onto a uniform background and linear adjustments of the contrast level of the entire image were made using Photoshop (Adobe; San Jose, CA). The higher magnification, single field of view images of paraffin sections were taken using the LAS multifocus software feature to combine 3–5 optical planes in a single extended depth of focus image and then an identical linear adjustment to the contrast level of the entire image was made to each of the images.

Wide-field fluorescence microscopy was performed on 0.5 μm acrylic resin cross-sections. No image processing steps were performed on fluorescent images other than cropping or re-sizing images to fit the journal requirements and to add text.

### Tissue preparation for transmission electron microscopy (TEM)

Specimens were fixed for greater than 24 hrs in 2% paraformaldehyde in HWB, rinsed with HWB, and further fixed using the osmium-thiocarbohydrazide-osmium technique [19]. Briefly, the tissues were exposed to 1% osmium tetroxide in HWB for 1 hr at room temperature, rinsed extensively in deionized water (dH_2_O), incubated in freshly prepared 0.4% thiocarbohydrazide for 30 min, rinsed in dH_2_O, and re-incubated in 1% osmium tetroxide for 1 hr. The second exposure to osmium caused a significant blackening of the tissue and resulted in superior contrast in the electron microscope. After further dH_2_O rinses and dehydration with an ethanol series, tissues were embedded in an epoxy resin (EmBed-812; Electron Microscopy Sciences, Fort Washington, PA). Thin sections were counterstained with uranyl acetate and Reynold’s lead citrate solution prior to viewing using a JEOL 1400 transmission electron microscope. The Photoshop Unsharp Mask Filter (125%, radius 3, threshold 3) was applied to all digital electron micrographs.

## Results

**Fig 1:**
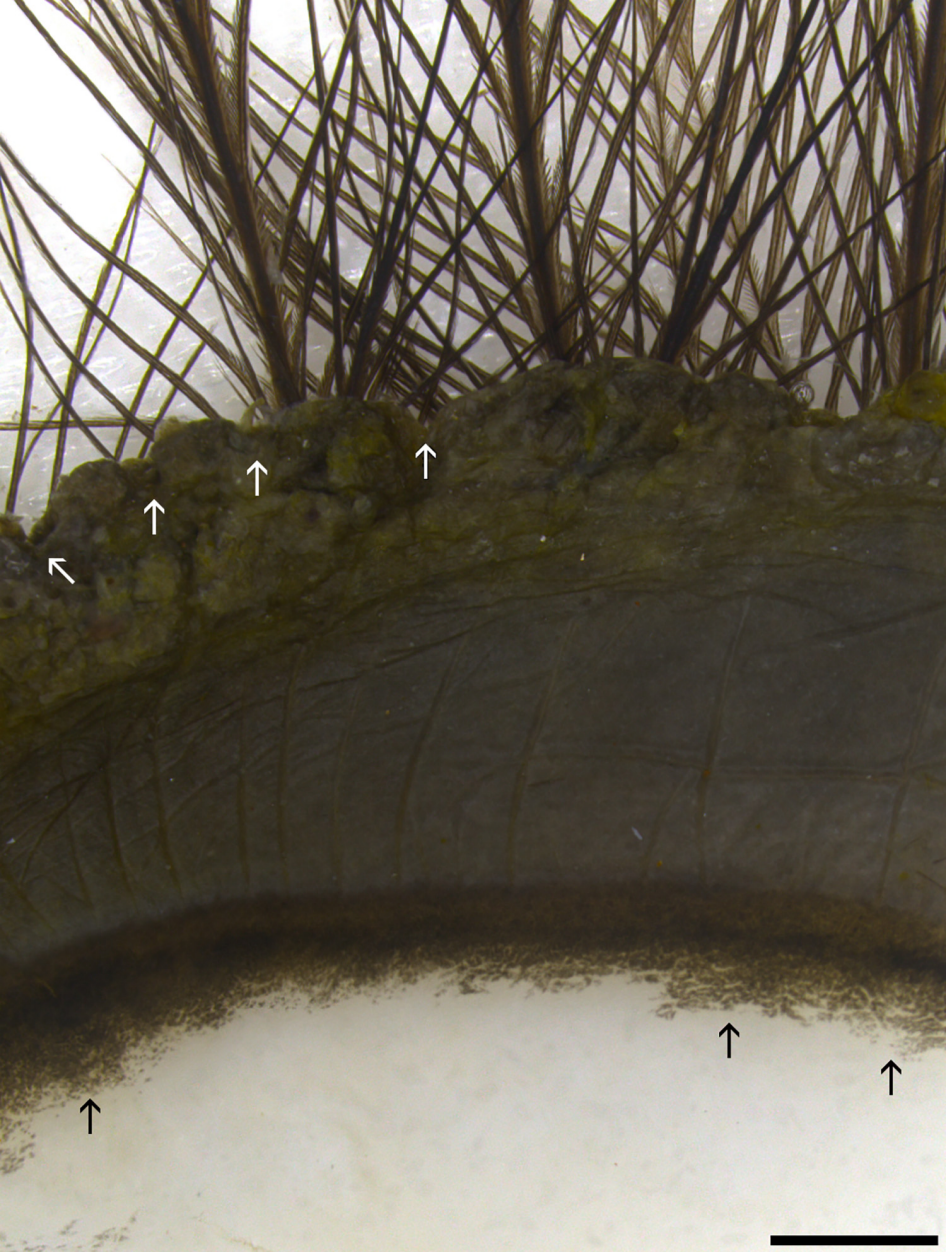
Marginal region of superior eyelid. The outer marginal edge has a scalloped appearance (delineated by tips of white arrows). Approximately the outer 2/3 of the marginal zone has heavy melanization before it makes a gradual transition to the melanin-free epithelia (black arrows). Hair-like filoplume feathers with few barbs are seen arising from the epidermal side of eyelid along the marginal edge. Bar = 1000 μm.

As is typical in birds, the lower eyelid was larger and more mobile than the superior eyelid. The outer marginal edge of each eyelid was deeply pigmented and puckered into “sausage-like segments” (Fig 1) similar to those first described in the English sparrow [20]. Filoplumes were present at the palpebral margin.

**Fig 2:**
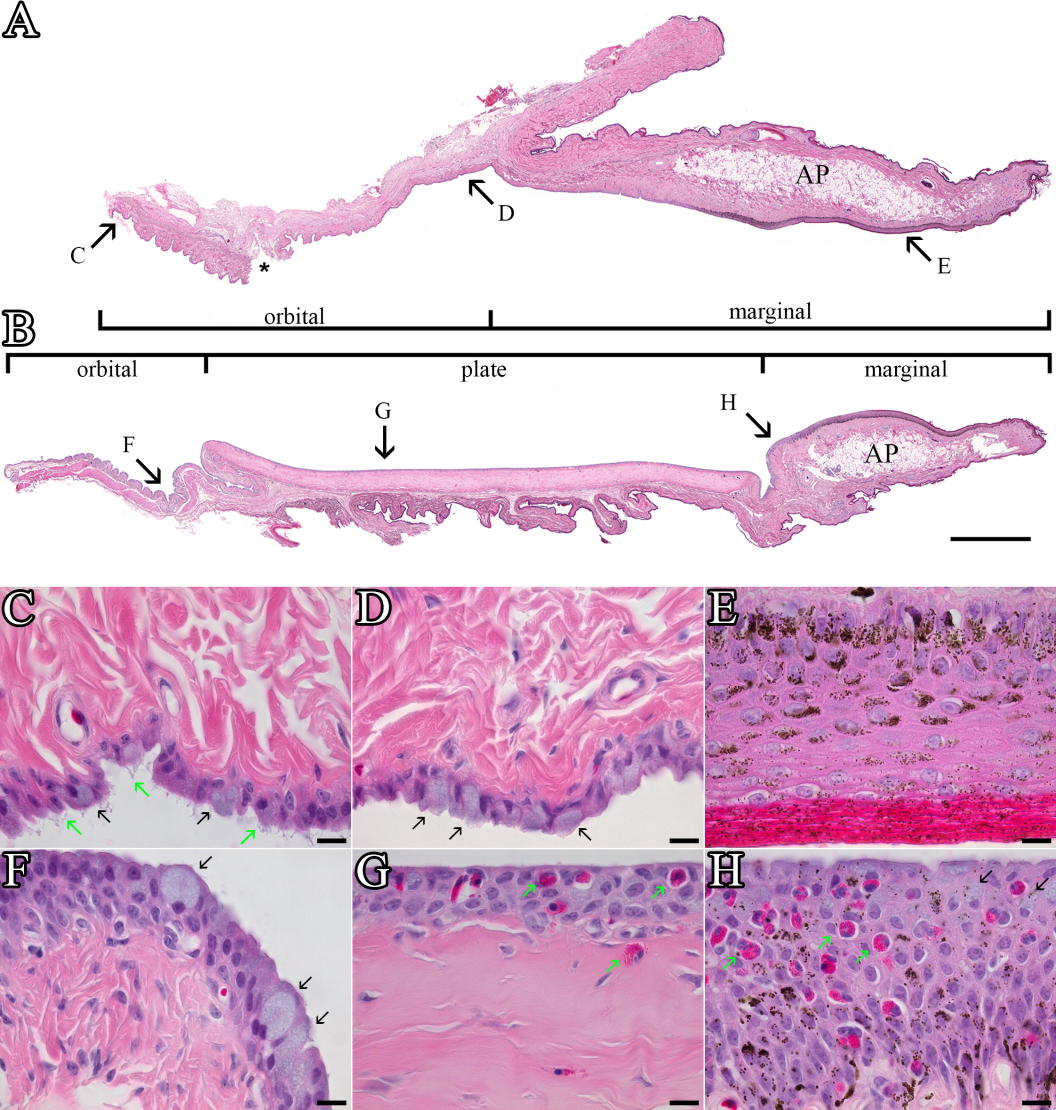
Superior and inferior eyelids. (A) Panoramic view of the superior conjunctiva created using 51 overlapping images of an H&E-stained paraffin section. A physical tear in the mucosa that occurred during processing is marked by an *. Bar = 1000 μm. (B) Inferior conjunctiva panoramic composite formed from 62 images. Large adipose pads (AP) are present in the center of the marginal zone of both the superior and inferior conjunctiva. The arrows labeled C through H on Figs 2A and 2B show the approximate location of the high power magnification views shown in Figs 2C through 2H. Bar = 1000 μm. (C) Distal orbital zone of the superior conjunctiva. Goblet cells (black arrows) are interspersed amongst epithelial cells with cytofilia extensions (green arrows) similar to those more extensively expressed in the third eyelid. Bar = 10 μm. (D) Proximal orbital zone of superior conjunctiva. The goblet cells (black arrows) in this region are intermixed with columnar epithelial cells that lack the cytofilia seen in more distal regions. Bar = 10 μm. (E) Marginal zone of the superior conjunctiva. The thick stratified squamous epithelium has a thick keratinized coat. Melanosomes can be observed in cells throughout the full thickness of the epithelium and into the keratinized layer. Bar = 10 μm. (F) The orbital zone of the inferior conjunctiva is typically more folded than the superior region. Plump goblet cells (black arrows) filled with mucous granules are scattered throughout the epithelium. Bar = 10 μm. (G) The plate region of the inferior conjunctiva is a stratified cuboidal epithelium with fewer goblet cells than seen in the orbital zone. It was not unusual to see granulocytes (green arrows) with eosinophilic granules within the epithelial layer or in the connective tissue just below it. Bar = 10 μm. (H) In the distal quarter of the marginal zone, the epithelium remains stratified squamous but is no longer keratinized as typically seen in the proximal marginal zone. The number of melanosomes present in the epithelium begins decreasing as the epithelium gets closer to the plate region. Goblet cells (black arrows) can be found in this region but are not generally found more proximally. Granulocytes (green arrows) are also found in the distal marginal zone but they are not typically as abundant as they are in the plate region. Bar = 10 μm.

The keratinized epidermis adjacent to the eyelid margin was relatively thin with typically 2–3 layers of nucleated cells. The epidermis in this region contains sparse numbers of melanocytes and is lightly pigmented. As the epithelium turns inward to form the palpebral conjunctival surface, it became much thicker with up to 10 layers of nucleated cells; the number of melanocytes and level of pigmentation increased dramatically (Figs 1, 2E & 2H). A large oval-shaped plate region dominated the middle half of the inferior eyelid (Fig 2B). The fibrous plate was slightly elevated from the surrounding palpebral epithelium and a shallow sulcus formed a moat-like region around it. No corresponding plate region was present in the superior eyelid.

The eyelid’s marginal zone remained keratinized for the approximately half the distance to the plate region and then tapers down to 5–6 non-keratinized layers before blending into the plate epithelium (Fig 2). There were relatively few secretory cells in the marginal epithelium but lectin staining identified occasional surface cells filled with intensely-stained vesicles consistent with a minor role in mucin secretion. The substantia propria underlying the epithelium of the eyelid margin contained a large number of non-descript mononuclear cells along with occasional melanocytes and granulocytes embedded in a dense fibrous mesh. The middle of the marginal zone, in both the inferior and superior eyelids, contained a large adipose pad as previously described in some but not all birds [20]. No glands were present in either eyelid’s marginal zone.

**Fig 3:**
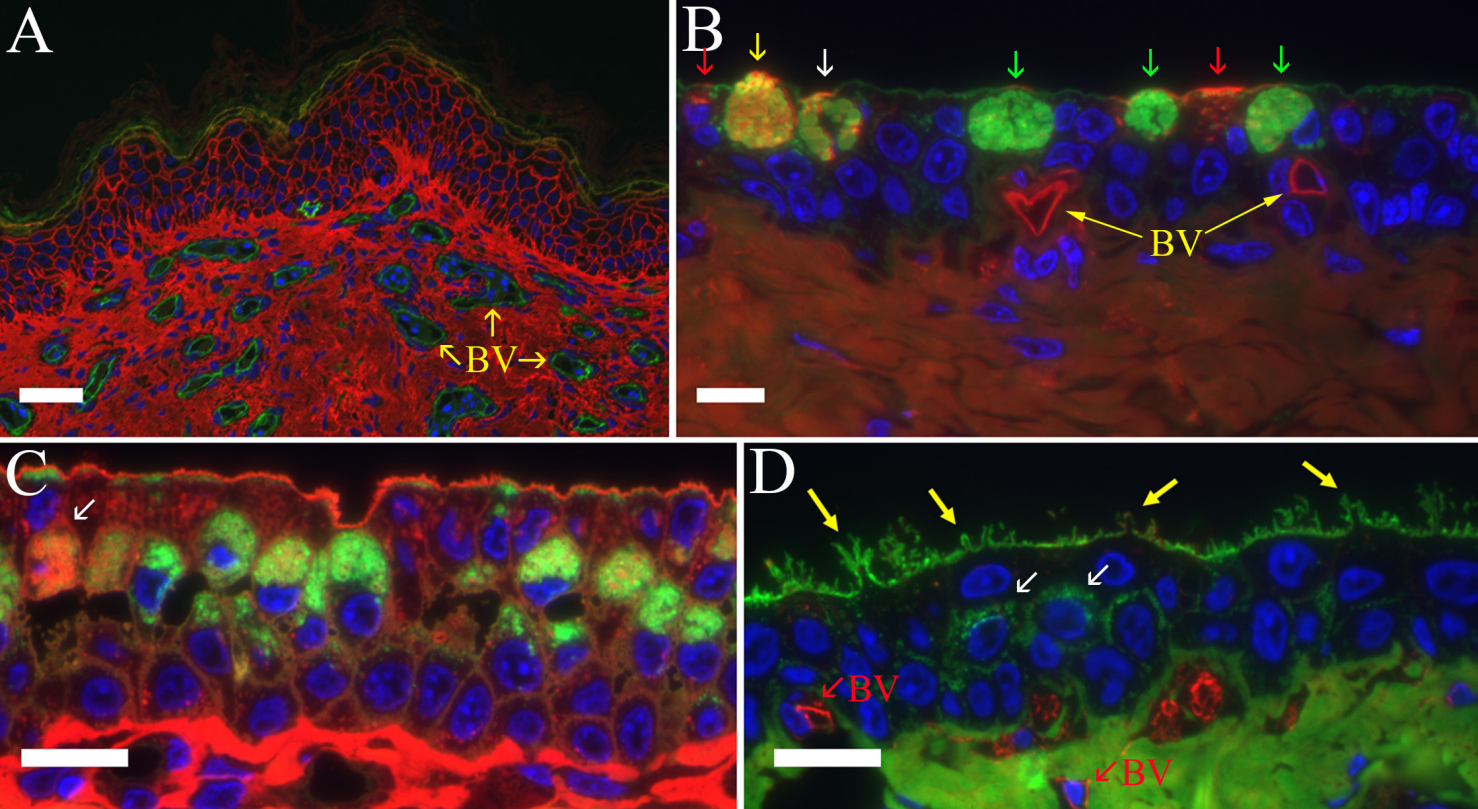
Lectin labeling of inferior conjunctiva. (A) Proximal marginal zone of an inferior eyelid. The lectin MAL-I (red) has stained the collagen and epidermal cell membranes. The endothelial cells lining small caliber blood vessels (BV) were stained by SBA (green). In all fluorescent images, the nuclei have been stained with DAPI (blue). Bar = 30 μm. (B) Orbital epithelium in the inferior conjunctiva stained with WGA (green) and VVA (red). The labeling pattern show in this image illustrates the difficulty in quantifying lectin staining of mucous secretory glycoproteins. Three plump goblet cells are predominately stained by only WGA (green arrows) while the secretory granules in another goblet cell stain equally with WGA and VVA (yellow arrow) and another cell has a mix of WGA + VVA-positive granules interspersed with WGA-only stained granules (white arrow). Finally, two cells with smaller concentrations of secretory vesicles are predominately stained only by VVA (red arrows). VVA also diffusely stains collagen in the underlying lamina propria and vividly stains the endothelia of two capillaries (BV) at the base of the epithelium. Bar = 10 μm. (C) The fibrous plate epithelium in barred owl #3 showed an unusually high number of goblet cells compared to the other birds. The goblet cell on the far left (arrow) is filled with secretory granules that are predominately LCA-positive (red) with a few distinct granules stained by WGA (green). Neighboring goblet cells are predominately labeled by WGA (green) with only a few LCA-stained granules. The WGA and LCA-stained granules appeared to be distinct populations within single cells rather than being a mixture of the two binding sites in a single granule such as more commonly seen with other lectin pairs. Note also that the most superficial layer of epithelium has relatively few glycoprotein-filled secretory granules consistent with rapid secretion of the mucin glycoproteins once the cell reaches the surface. The apical surface of the superficial layer is much flatter and only has a limited number of short microvilli compared to the plates shown in Figs 3D and 4B. Bar = 10 μm. (D) Plate epithelial cells showing cytoplasmic extensions (yellow arrows) of the apical membrane similar to those found on the third eyelid’s bulbar surface. WGA (green) stains the apical surface of the superficial layer, a few secretory granules in the mid-zone of the epithelium (white arrows), and the collagen in the underlying connective tissue. GSL isolectin B4 (red) stains only the endothelial cells of small capillaries (BV) at the base of the epithelial layer. Bar = 10 μm.

**Fig 4:**
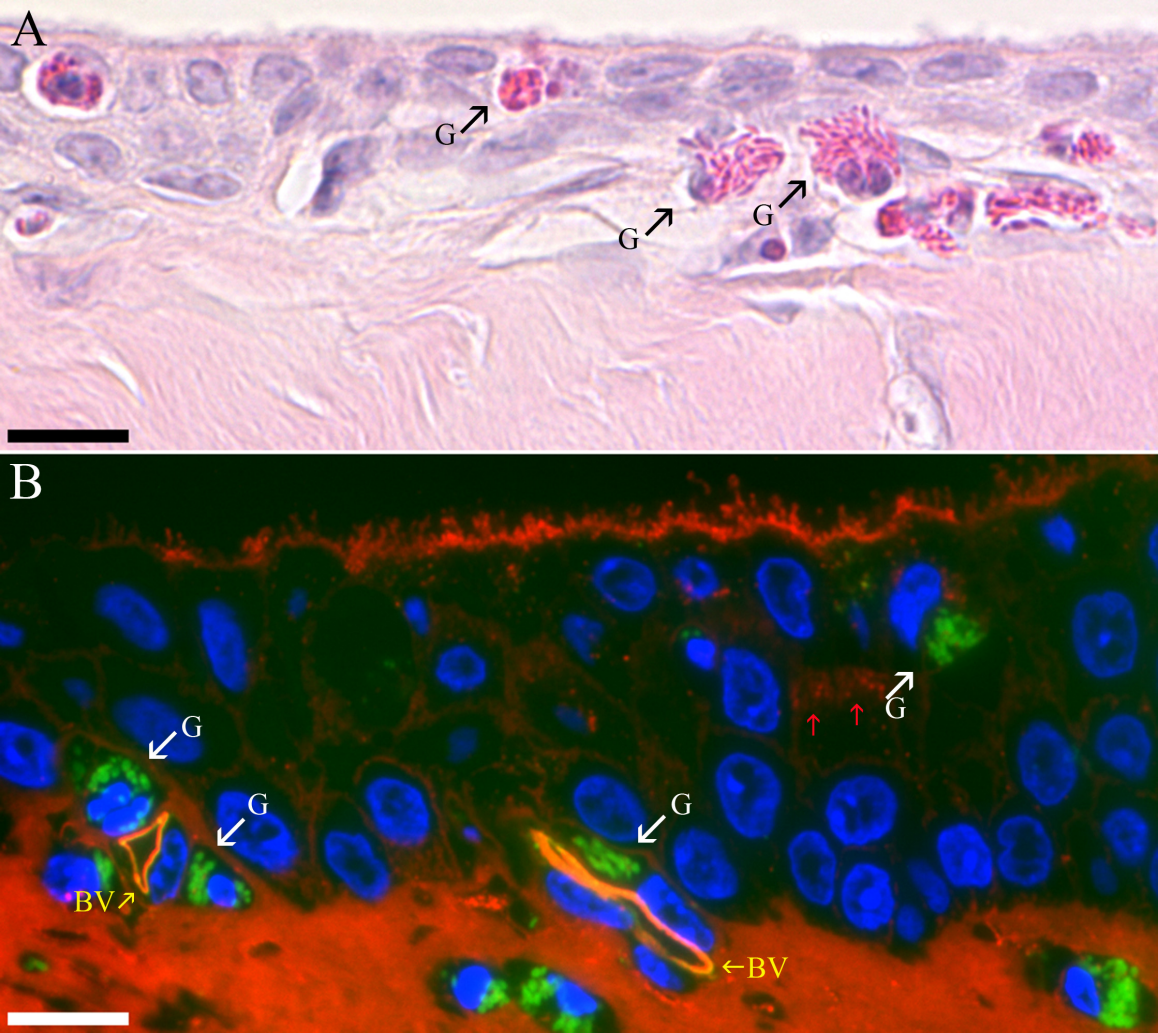
Granulocytes in the plate region. (A) Granulocytes (G) with bright red secretory granules can be seen penetrating the epithelia layer of the fibrous plate in an H&E stained paraffin section. Bar = 10 μm. (B) Plate epithelium stained with a combination of LEL (red) and GSL-I (green). GSL-I intensely stains the secretory granules of granulocytes (G) in the plate epithelial layer and underlying connective tissue. LEL stains the collagen, microvilli and a few secretory granules in a cell (red arrows) located in approximately the middle layer of the stratified plate epithelium. The microvilli on the surface of these plate epithelial cells are short stubby compared to those in the plate regions illustrated in Fig 3C and 3D. Endothelial cells lining blood vessels (BV) are stained by both LEL and GSL-I. Bar = 10 μm.

The endothelial cells lining conjunctival blood vessels were readily labeled by a variety of lectins including those with specificity against N-acetylgalactosamine (e.g., SBA in Fig 3A; VVA in Fig 3B), galactose (e.g., GSL-I Isolectin B4 in Fig 3D or GSL-I in Fig 4B), or N-acetylglucosamine (LEL in Fig 4B). The rich vascularity of the eyelid margin was easily appreciated when fluorescein-tagged SBA was used to label the endothelial cells against a surrounding background of collagen stained red by using MAL-I-Alexa568 (Fig 3A).

**Fig 5:**
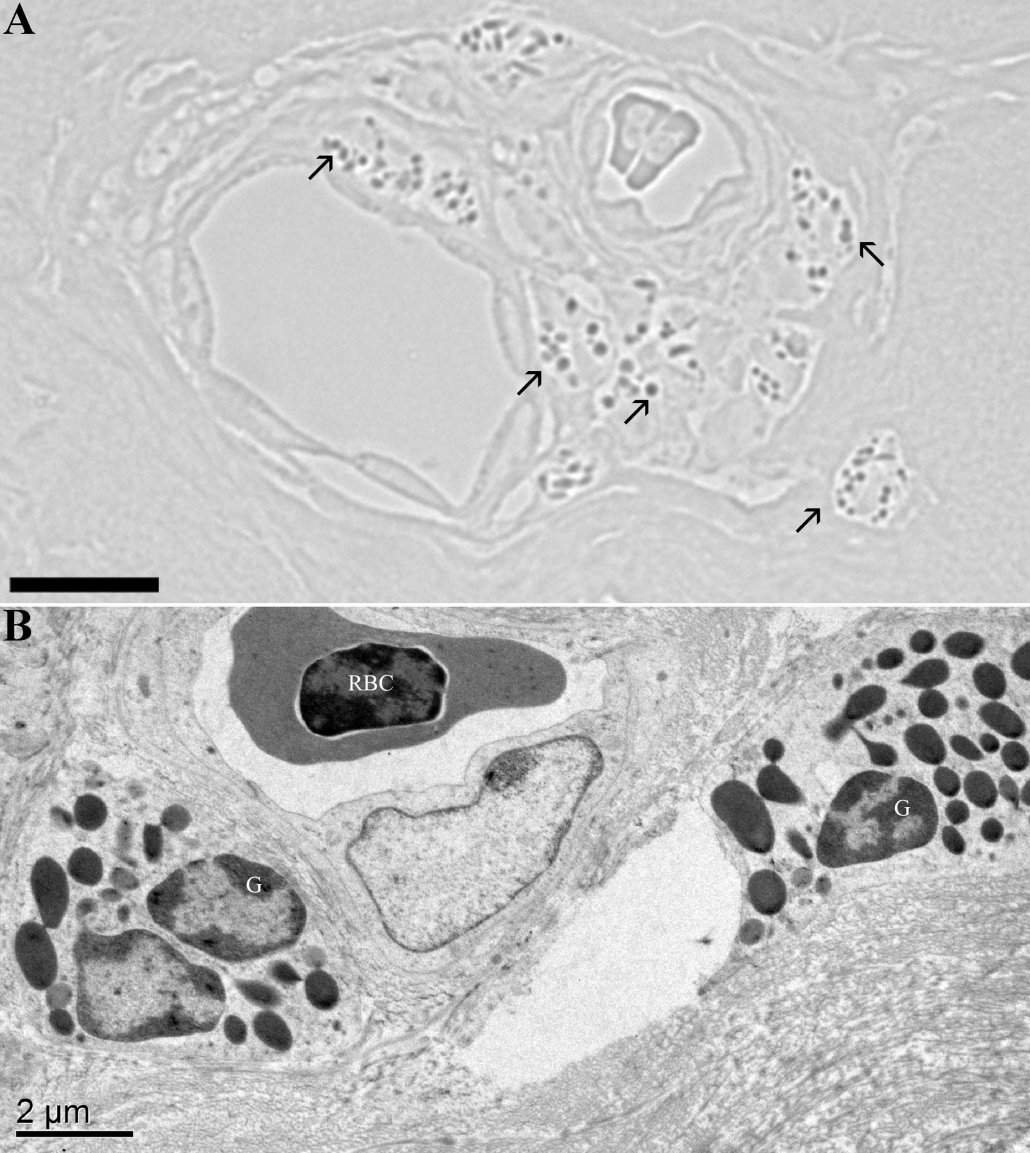
Eosinophils. (A) Peroxidase-positive reaction in granules (arrows) of granulocytes in a perivascular cuff demonstrates these cells are eosinophils. Bar = 10 μm. (B) A transmission electron microscopic view of a similar perivascular cuff of granulocytes (G) surrounding a small blood vessel in orbital zone of the inferior conjunctiva. A nucleated red blood cell (RBC) is present in the vessel. Bar = 2 μm.

The fibrous plate region was covered by a non-keratinized epithelium that varied from stratified squamous to stratified cuboidal within a given inferior eyelid (Figs 2G, 3C, 3D & 4). The thickness of the epithelium also varied within a plate region between 2 to 7 layers but was typically 3 to 5 layers (Fig 2G). The apical membrane of the most superficial layer of cells was in some cases fairly flat (Figs 2G & 3C) while other regions had long microvilli (Fig 4B) and even occasionally contained apical membrane extensions similar to those routinely seen in the third eyelid (Fig 3D). The plate regions in all of the birds contained at least a few plump epithelial cells filled with mucous secretory granules as well as many other cells containing smaller numbers of glycoprotein-filled vesicles stained by the lectin probes (Fig 4B) but the relative proportion of well-differentiated mucous cells was much lower than in the orbital conjunctiva. The plate region in bird #3 had significantly more cells with large accumulations of mucous secretory granules (Fig 3C). The most superficial epithelial layer frequently appeared to have fewer granules stained by lectins suggesting that the glycoproteins seen in deeper layers were rapidly secreted once cells migrated to the surface. A thick dense fibroelastic stroma filled the substantia propria directly below the epithelium. Capillaries were often observed directly applied against the epithelium (Fig 4B). One unexpected finding in all six birds was the large number of granulocytes located in either perivascular rings (Figs 4B & 5), just below the epithelium (Fig 4B) or even infiltrating an otherwise healthy-looking epithelium (Fig 2G). The granulocytes were easily recognized by their bright eosinophilic granules in paraffin sections (Fig 4A) or intense labeling by GSL-I in the fluorescence images (Fig 4B). GSL-I is a mixture of A and B subunits that combine to form five tetrameric isolectins with a mixed binding specificity for α-galactose and α-N-acetylgalactosamine. The GSL-I-B_4_ isolectin, made up of only B subunits, has a more selective binding specificity of only α-galactose and did not effectively label the granulocytes consistent with granule-staining being due to α-N-acetylgalactosamine-rich targets. The granules in these cells had a positive peroxidase reaction which demonstrates the cells are eosinophils since avian eosinophils, but heterophils, are known to be peroxidase-positive [21] (Fig 5A).

The palpebral surface of the orbital zone in both the superior and inferior eyelids had an irregular surface with mucosal pleats that presumably flatten out as the eyelid extends across the globe (Fig 2). Well-differentiated goblet cells were abundant (Fig 2C, 2D & 2F). Lectin staining revealed considerable heterogeneity in the glycosylation of the mucin secretory product (Fig 3B). Although many cells in the epithelium had vesicular glycoproteins stained by the lectins, the following observations are focused on staining of secretory granules in well-differentiated mucous cells with classic goblet-shaped morphology. The N-acetylglucosamine-binding lectin WGA came close to being a probe able to homogeneously label all goblet cells although the intensity of labeling varied between cells. All other lectins listed in Table 2 were much less effective in labeling goblet cells. Generally no more than 50% of the goblet cells, and often significantly fewer, showed any positive labeling of secretory granules by individual lectins. It is difficult to be more precise in quantifying lectin-positive staining since adjacent columnar cells often had smaller amounts of lectin-stained glycoprotein-filled vesicles which confounded differentiating true goblet cells from columnar cells that were members of the so-called “second mucous system” previously described in the conjunctiva [22]. Quantitative assessment was further hindered by the observation that even in the plump goblet cells, the staining pattern was often heterogeneous with some granules labeling intensely while there was little or no staining of adjacent granules in the same cell. Granulocytes, similar to those seen in the plate region, were sometimes observed in the orbital zone but less commonly than in the fibrous plate.

Organized conjunctiva-associated lymphoid tissue (o-CALT) was observed in three of the six birds (not shown). Single lymphoid follicles were found pressed up against the orbital epithelium but not in more proximal regions of the palpebral conjunctiva nor in the third eyelid. Lymphoid cells from the follicle penetrated into the overlying follicle-associated epithelium (FAE) and filled basolateral pockets between epithelial cells. Goblet cells were excluded from the FAE.

**Fig 6:**
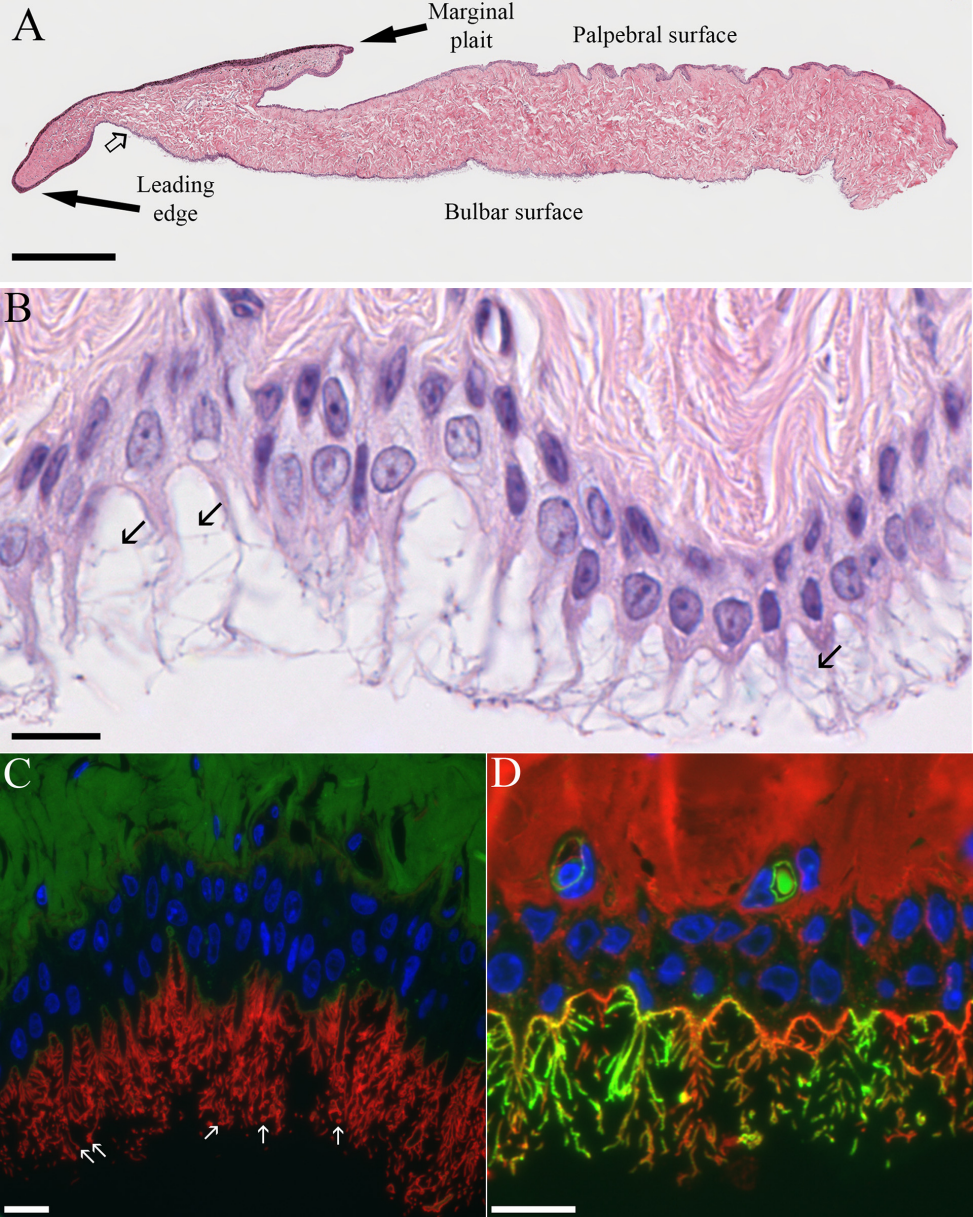
Light microscopic views of the third eyelid. (A) Panoramic composite view of H&E-stained paraffin section of third eyelid made from 14 images. The point where the smooth stratified epithelium on the bulbar surface first begins to show cytoplasmic extensions is marked by the open arrow. Bar = 500 μm. (B) Bulbar surface of the third eyelid illustrating cytoplasmic extensions with lateral cytofilia (arrows) coming off of them. Bar = 10 μm. (C) The apical membrane near the base of the cytoplasmic extensions is only labeled by MAL-I (green) while the membrane of the extensions and cytofilia are intensely labeled by ECL (red) Arrows point to circular profiles of the bulbous ends of the cytofilia. Bar = 10 μm. (D) Even when a cocktail of WGA, SBA, DBA, PNA and SBA (all red) are used, some cytofilia are more strongly labeled by GSL-I (green). The relative absence of lectin-stained secretory granules in Figs 6C and 6D underscores the negligible contribution of this epithelium to the mucin component of the tear film. Bar = 10 μm.

**Fig 7:**
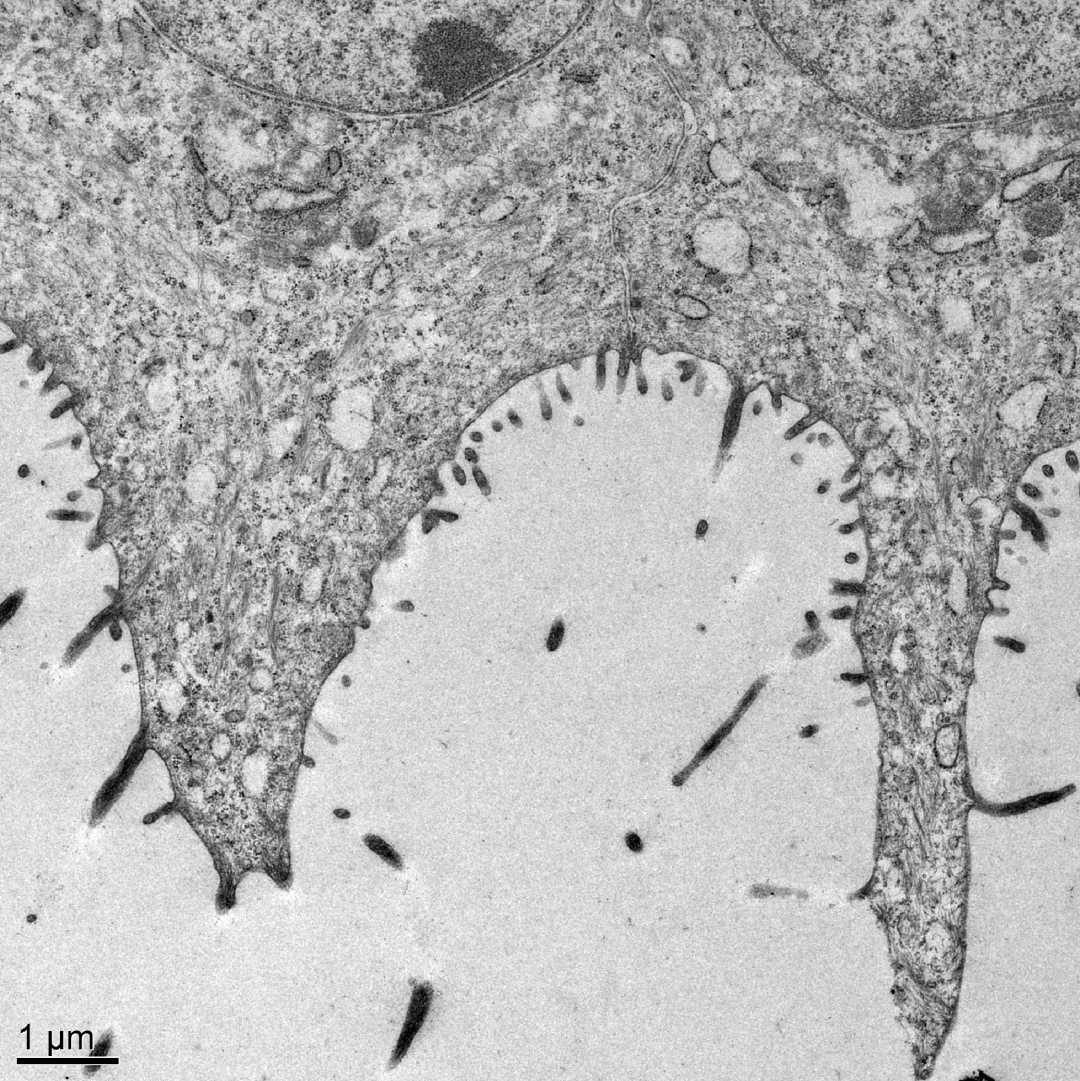
Electron microscopic view of cytoplasmic extensions and cytofilia on bulbar epithelium of the third eyelid. The cytoplasmic extension coming off the cell on the left spans from one margin of the cell to the next. The adjacent cell’s cytoplasmic extension is much narrower and involves only the central third of the apical membrane. Bar = 1 μm.

**Fig 8:**
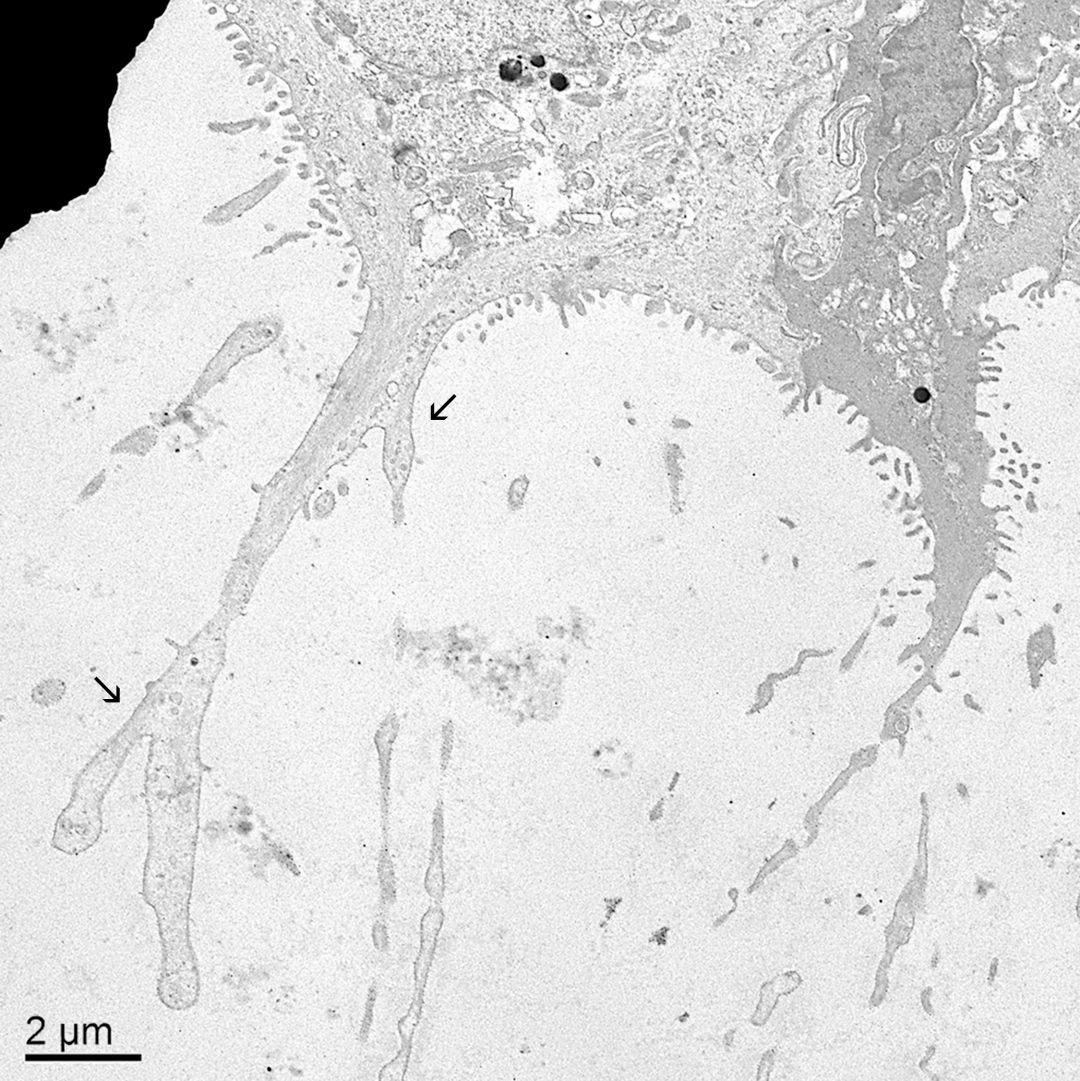
Electron microscopic view of branching, irregular cytoplasmic extensions and cytofilia. The diameter of the cytoplasmic extensions and cytofilia were often irregular. Cytofilia frequently branched (arrows). Note the relative absence of secretory vesicles in the cytoplasmic extensions. Bar = 2 μm.

The tapered leading edge of the third eyelid was covered by a pigmented, non-keratinized stratified squamous epithelium. The palpebral surface of the third eyelid was covered by an approximately five-cell-deep stratified squamous epithelium. Lectin staining did not reveal any glycoprotein-filled vesicles in these cells except for an occasional mucous cell near the junction with the superior or inferior eyelids. On the bulbar surface of the third eyelid, the pigmented, stratified squamous epithelium soon turned into an unpigmented, bilayer of epithelial cells. It was the apical membrane of the superficial cells in this bilayer that gives rise to the distinctive “feather duster” architecture of the third eyelid. The structure of the apical membrane of these cells was so unique that it is difficult to describe it in terms normally used to characterize other epithelia. The apical surface of the cells was occasionally flat but more commonly formed domes or had elaborate protrusions (Fig 6). The domes were simply a bulging apical membrane similar to what might be seen in a transitional epithelium of the urinary tract. In many cases, however, the apical membrane extended out as far as 25 μm in narrow, tapering extensions. The cytoplasmic extensions sometimes involved the entire span of the apical membrane from one cell margin to the next but could also be restricted to the middle third or half of the apical surface with the adjacent membrane on either side being relatively flat (Figs 7 & 8). The apical membrane was covered by short, conventional microvilli that were typically 80–100 nm wide and less than 0.5 μm long (Figs 7 & 8). Unlike the even coat of microvilli that might be seen in the brush border of the intestine, the density of microvilli on the bulbar surface was less than 50% of the available space and when the cell formed the long extensions, microvilli were less common in the more apical regions. Interspersed amongst the microvilli were narrow, more irregular cellular processes known as cytofilia (Figs 7 & 8). Cytofilia extended 15–20 μm outwards from both the cytoplasmic extensions and flat stretches of the apical membrane. There was often a bulb or swelling at the end of the cytofilia (Fig 6C). The width of the cytofilia was much more irregular than one would see in a microvillus or stereocilium and typically ranged from 150 to 300 nm but occasionally bulged out to 800 nm. The cytoplasm of the cytofilia had occasional vesicles but no consistent filamentous core such as would be seen in stereocilia or cilia. The distinction between cytoplasmic extensions and cytofilia could be subtle at times. The intensity of lectin staining of the membranes of the cytoplasmic extensions and cytofilia was often different from that in the flatter regions of the apical membrane adjacent to the cytoprotrusions (Fig 6C). Likewise, the lectin staining of cytofilia on adjacent cells were often markedly different (Fig 6D). Even when a cocktail of five lectins was used to stain the nictitating membrane, there was no evidence of goblet cells filled with mucous granules in any of the birds except for occasional cells in bird #3.

## Discussion

The normal anatomy of the owl (Strigiformes) eye has been well-described [2]. The histology of the eyelids and third eyelid, on the other hand, has largely been unexplored except for some passing mention in the early literature [3, 23]. The present study is the first histological and fine structure characterization of the upper and lower eyelids, as well as the third eyelid, of the barred owl.

As previously described in other birds, no sebaceous glands were observed in either eyelid. This is consistent with avian skin in general not having sebaceous glands but rather the epidermal epithelium as a whole producing the phospholipids and neutral fats usually produced by those glands [24]. Whether the avian conjunctival epithelium similarly produces lipids that fulfill the anti-evaporative role in the tear film that are ascribed to lipids produced by meibomian glands in mammalian eyelids remains unknown. Lipid secretion from the Harderian glands may also fulfill this role in the barred owl.

The palpebral conjunctiva in mammals is generally described as having marginal, tarsal and orbital zones but more appropriate terminology in raptors would be marginal, plate and orbital zones. The marginal zones of both eyelids were highly vascularized which is consistent with an earlier report that small-caliber palpebral arteries are particularly prone to injuries resulting from lacerations [2]. There is limited information on histology of the inferior plate region in owls, falcons, hawks or vultures. One study in the common buzzard (*Buteo buteo*) described the dense meshwork of collagenous fibers in this region but did not discuss the superficial epithelial layer [25]. In the barred owl, the plate was found to be covered by a stratified epithelium with relatively modest numbers of well-differentiated goblet cells. Mucin glycoproteins are another essential component of the tear film but there are surprisingly few mucous cells in the marginal and plate epithelium in 5 of the 6 birds. The higher number of goblet cells in bird #3 may have been a reactive change since this was the only bird whose clinical history included significant ocular disease. Whether the absence of sebaceous glands and relative scarcity of goblet cells affects tear film stability or is linked in some way to the unusual apical membrane specializations of the bulbar surface of the third eyelid remains unknown.

The anatomy of the third eyelid is well-suited for its proposed role in cleansing the eye’s surface of debris. As it passes over the eye, fluid and particulate matter presumably flows across the superficial surface of the leading edge until it passes over the marginal plait, which is a fold or pleat of tissue that runs along the length of the free margin. Fluid and debris in the sulcus formed by the junction of the marginal plait with the third eyelid’s superficial surface would be then carried to the medial canthus and lacrimal ducts during retraction of third eyelid [20, 26]. The histology of the barred owl third eyelid was similar to earlier studies performed using the pigeon. The bulbar surface of the pigeon nictitans has been described as pseudostratified while the barred owl is a true stratified cuboidal or columnar epithelium with a consistent basal row of cuboidal cells [5, 6] The first report of the elaborate epithelial apical membrane specializations on the pigeon nictitans described the superficial cells on the bulbar surface as having conically tapered cytoplasmic extensions that extended up to 25 microns from the apical surface before ending in a small club or lobe [3]. Furthermore, the central extension was described as having numerous fine extensions measuring up to 5 microns that gave the structure the appearance of a small feather or spruce branch [3]. Later studies referred to the lateral extensions as cytofilia [5, 6]. A scanning electron microscopy study of the pigeon nictitans confirmed the central region of the bulbar surface was covered with branching apical outfoldings that looked like small trees or feather dusters [6]. The authors noted that the lateral processes off the cytoplasmic extensions always ended in a spherical or button-shaped bulge. The LM and TEM studies of the present paper demonstrate the central extensions on the barred owl are similar in overall morphology and length to those described in the pigeon but that the cytofilia coming off the barred owl extensions were 4–5x longer than those in the pigeon. Spherical bulges were also observed at the ends of most barred owl cytofilia. A TEM study of the pigeon nictitans found no evidence of a fibrillar core in the cytoplasmic extensions or cytofilia and concluded they must be non-motile [4]. Similar observations in the present study would indicate the owl cytofilia are also non-motile. Two groups reported that bulbar epithelium of the pigeon nictitans as having variable amounts of secretory vesicles that was considered consistent with a role in mucin or lipid secretion [5, 6]. Both of these papers referred to histochemical staining of the mucins but neither showed images or data supporting these findings. In one of the papers, the TEM images clearly showed the cytoplasmic extensions were filled with more secretory granules than typically seen in the barred owl tissue [5]. Although occasional goblet cells were observed on the bulbar surface of the owl’s nictitans, its role in producing the mucin component of the tear film would seem to be limited.

The apical membrane specialization of the third eyelid’s bulbar epithelium has an interesting phylogenic distribution. At the base of the avian tree of life, the Paleognathae (raites and tinamous) are a distinct radiation from the Neoganathae [7, 8]. The Neoganathae then split into the Galloanserae (including chickens and ducks) and the Neoaves (all other species). The land birds are the largest clade within the Neoaves and includes owls, hawks, falcons, New World vultures, and the Passeriformes or perching birds. There is general agreement that pigeons (Columbiformes) lie in a Neoave clade distinct from the land birds although it is controversial whether they are part of a larger clade known as the Metaves. The present study shows the unusual apical membrane specialization of the third eyelid that has been previously well-characterized in the Columbiformes is shared by the barred owl, a member of the land bird clade. Interestingly, the membrane specialization is lacking in a penguin species that is a member of the water bird clade within Neoaves [27]. The membrane specializations are also absent in two species of ducks within the Galloanserae [27]. The rockhopper penguin, hooded merganser, which is a diving duck species, and the common mallard, which is a dabbling duck species, all have third eyelids with similar gross anatomy as the pigeon and barred owl but relatively smooth apical membranes on their bulbar epithelial surface; electron microscopy found only a few short microvilli on their bulbar epithelium [27]. Whether the similarity of the third eyelid morphology in the rockhopper penguin and two duck species is related to their water habitat or reflects some other physiological adaptation awaits examination of third eyelid histology in a wider range of bird species. It is also not possible to use the present results to establish that a nocturnal species has similar third eyelid specializations as those previously described in diurnal species since the barred owl is not strictly nocturnal. Barred owls are generally described as crepuscular/nocturnal hunters active at both twilight and night and there have even been reports of diurnal hunting [28].

Organized conjunctiva-associated lymphoid tissue (o-CALT), consisting of a well-developed lymphoid follicle in intimate contact with palpebral epithelium, has previously been described in chickens, turkeys, and mammals such as humans, rabbits, and Guinea pigs [29–32]. The follicle-associated epithelium is typically devoid of goblet cells and has been shown to contain the distinctive M cell which is capable of translocating proteins, latex beads and bacteria [33]. M cells are believed to transport soluble and particulate antigens to the underlying follicle as the first step in generating a mucosal immune response. In chickens and turkeys, extensive arrays of o-CALT develop in the lower eyelid, and to a lesser extent in the superior eyelid, soon after hatching [32, 34]. The o-CALT seen in the barred owl consisted of single follicles pressing up against the epithelium in the orbital region. Although o-CALT is often found in the mammalian third eyelid [35, 36], no examples were observed in this region of the barred owl. In other species, third eyelid follicles are typically closely apposed to the bulbar surface epithelium and associated with the specialized antigen-sampling M cell in the epithelial layer. The elaborate membrane specializations of the bulbar surface epithelium of the barred owl third eyelid may preclude o-CALT in this region.

The consistent presence of granulocytes in the conjunctiva was an unexpected finding. Differentiating eosinophils from heterophils in routinely stained sections can be difficult in birds but the histochemical demonstration that the granules in these cells were peroxidase-positive established them as eosinophilssince avian heterophils lack peroxidases [21]. The role of eosinophils in birds is poorly understood but in mammals these cells are typically elevated in response to allergies or parasites. There was, however, no evidence of conjunctivitis or an ongoing infection that would explain their presence. It will be interesting to examine in future studies whether other species of birds of prey have similar elevations in conjunctival eosinophils.

Two of the six birds used in this study had ocular lesions noted in their initial clinical examinations (Table 1). The prevalence of ocular problems in this small sample set is consistent with earlier epidemiological reports. One retrospective study of free-living raptors admitted to either an academic veterinary clinic or raptor rehabilitation center found 14.5% of the birds had some type of ocular lesion; for barred owls in particular, the prevalence rose to 28% [37]. Of the 203 ocular lesions reported in this earlier study, 8.9% involved the accessory structures of the eye. The authors felt that their retrospective study may have been underestimated the true prevalence of ocular lesions since the cohort contained a large percentage of juveniles. In a colony of 23 Screech owls, 83% of the birds had at least one eye with an ocular abnormality [38]. A retrospective study of disorders of the third eyelid in birds that presented at three Ophthalmology Services at three major veterinary schools identified 17 birds, including 2 owls with traumatic injuries, 1 hawk with inflammation, 3 kestrels with dystrophic disorders, and 1 hawk with neoplasia [39]. The authors concluded that that disorders of the third eyelid are uncommon in birds.

The barred owl results in the present study form a foundation for future comparative studies with a phylogenic background that may give insight into the adaptive significance and evolutionary history of the morphological traits of the third eyelid and inferior fibrous plate in other raptors and more distant radiations.
